# Intrathoracic migration of ventriculoperitoneal shunt: a case report

**DOI:** 10.1186/1757-1626-1-42

**Published:** 2008-07-17

**Authors:** S Karapolat, A Onen, A Sanli

**Affiliations:** 1 Department of Thoracic Surgery, Faculty of Medicine, Dokuz Eylul University, Izmir, Turkey

## Abstract

**Introduction:**

Intrathoracic migration of ventriculoperitoneal shunt can be transdiaphragmatic or supradiaphragmatic. This complication causes important respiratory symptoms.

**Case presentation:**

A 7 year-old Caucasian female, hospitalized with the prediagnosis of pneumonia, was determined to have ventriculoperitoneal shunt migration at left hemithorax. A left thoracotomy was performed and the shunt was successfully removed transdiaphragmatically.

**Conclusion:**

The patients with intrathoracic migration of ventriculoperitoneal shunt must be treated surgically as soon as possible. Transdiaphragmatic surgical approach would be more suitable from the point of surgical easiness.

## Introduction

Complications of ventriculoperitoneal shunts may occur anywhere along their course, from the cerebral ventricle to the peritoneal cavity [1]. Among these potentials, intrathoracic migration of shunts is unusual and potentially serious [2]. The purpose of this paper is to report a case of intrathoracic migration of ventriculoperitoneal shunts because of its rarity.

## Case Report

**Figure 1:**
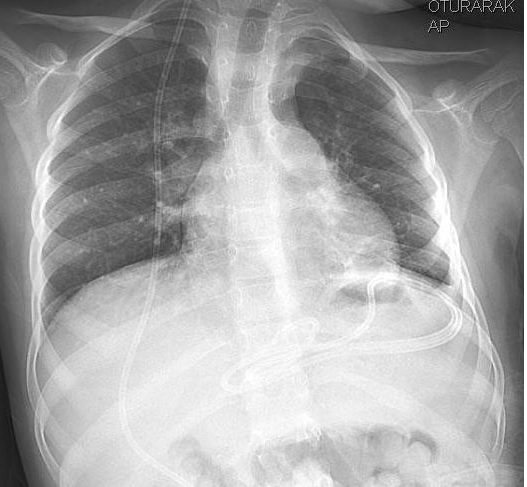
In chest roentgenogram, the ventriculoperitoneal shunt located in the left subdiaphragmatic region.

**Figure 2:**
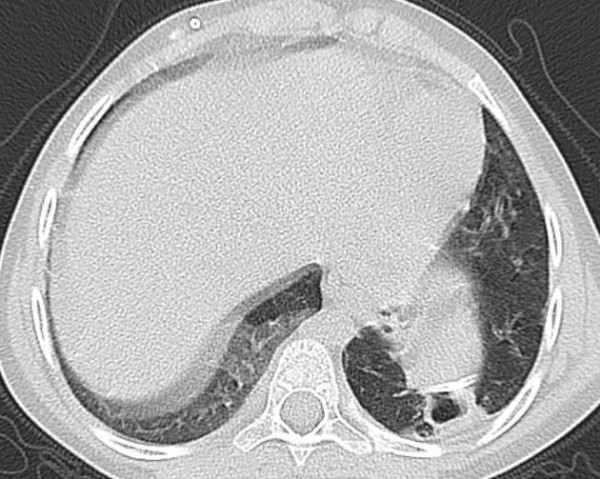
In computed tomography of thorax, the cavitary lesion with a diameter of 2 cm at lower lobe of left lung, and the catheter end at left hemithorax.

A 7 year-old Caucasian female was admitted to our hospital because of chest pain, cough, and fever. We learned from her medical history that, a ventriculoperitoneal shunt was placed with the diagnosis of hydrocephalus when she was 6 months old, and that the proximal part of the shunt was removed due to dysfunction two months ago but the part in the abdomen was left in its place and a new shunt was placed. During the physical examination, only crepitant rales were detected by chest auscultation. The chest roentgenogram showed an old shunt adjacent to diaphragm in left upper abdomen and a new shunt extending from in the right side (Fig. 1). Computed tomography of thorax revealed a cavitary lesion with a diameter of 2 cm at the lower lobe of left lung, bronchiectasic areas, partial pleural thickening and a catheter end in left hemithorax (Fig. 2).

**Figure 3:**
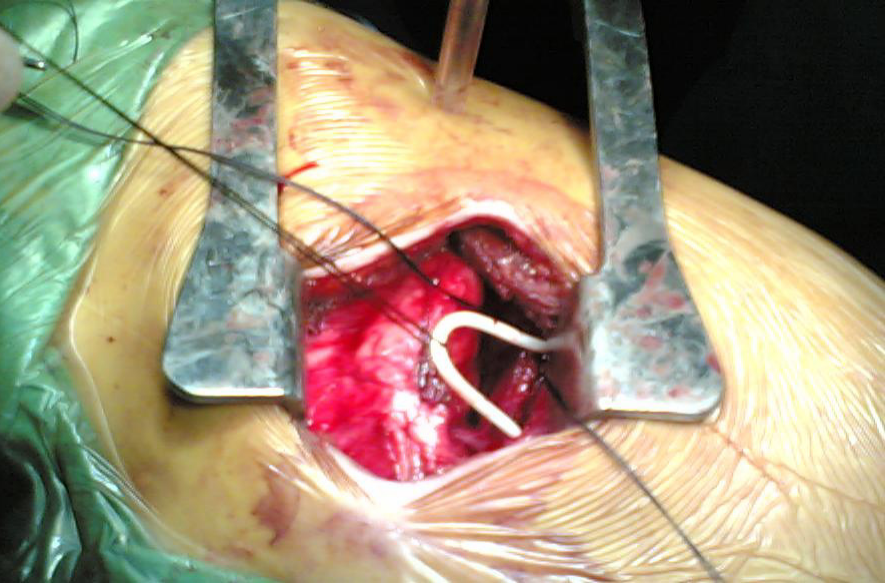
Removal of the shunt transdiaphragmatically during operation.

We performed a left thoracotomy from 6^th ^intercostal space and encountered a 1 cm defect in the middle of left diaphragm and the 4 cm end part of the catheter entering from this defect into thorax cavity. The borders of the defect in diaphragm were widened and the shunt was removed by eliminating the adhesions in the abdomen, and then the diaphragm was closed primarily (Fig. 3). After 5 days of postoperative period, patient was discharged from the hospital without any clinical problem. She was remained asymptomatic for 6 months follow-up after operation.

## Discussion

Inadequate catheter length and not fixing the shunt firmly enough to peripheral tissues are the main causes of shunt migration [3]. Mechanism of shunt migration is various. The catheter and located adjacent to diaphragm may slowly draw back into the thorax due to negative inspiratory pressure. Besides, the intraperitoneal fibrosis resulting from shunt may apply a continuous pressure to the upper part of the shunt adhered to the diaphragm and thus may cause a diaphragmatic perforation. The ventriculoperitoneal shunt can migrate through the diaphragm into the chest on its own or after an abdominal inflammatory process occurs. In addition, shunt may pass to the thorax by means of hiatus or congenital fenestrations in diaphragm. In our case, migration of ventriculoperitoneal shunt into chest was transdiaphragmatic. There was an open communication between the peritoneal and pleural cavities. We think that the local inflammation taking place around the shunt and subdiaphragmatic area as an occurrence mechanism may cause diaphragmatic erosion and perforation and the shunt prolapsed into the pleural cavity from this defect.

Intrathoracic migration of shunt may lead respiratory complications such as pleural effusion, pneumothorax, bronchial fistula, hydrothorax, empyema and pneumonia [4]. Our case showed a pneumonic situation.

Removal of the shunt surgically should be the main method for the treatment of this complication. After opening the diaphragm wide enough, the relationship among the abdominal cavity organs and especially the relationship of omentum with ventriculoperitoneal shunt should be examined carefully, and the adherences must be separated precisely and the shunt should not be pulled extensively and not stretched during these procedures. If shunt is pulled out without being free enough, the breaking possibility of it is high. In such a case, it will be difficult to remove the broken piece transdiaphragmatically from the abdomen. In our case, omentum surrounded the shunt from some separate parts and adhered firmly. Shunt could be removed only after separating thoroughly from omentum by a careful dissection.

In order to avoid such a complication, it should be paid attention not to keep the intraperitoneal part of the shunt very long. The more flexible material of shunt used is, the less the diaphragmatic perforation possibility will be. Besides, dysfunctional and unnecessary shunts should be taken out thoroughly. The shunts which are asymptomatic and need major surgery to be removed, and thus left in their places should be followed-up from the point of intrathoracic migration by means of radiographical examination at reasonable intervals [5].

## Conclusion

The treatment of this complication is to remove the shunt surgically as soon as diagnosed promptly. Thoracotomy should be made from a lower part than the classical incision place since it will be easier to reach the diaphragm. The removal of the shunt transdiaphragmatically would be more suitable from the point of patient's comfort and surgical easiness. However, as preventive actions, not keeping the shunt very long in the first application, and removing the dysfunctional shunts totally under elective conditions will prevent the occurrence of intrathoracic migration, and thus the unnecessary morbidity and mortality.

## Consent

Written informed consent was obtained from the patient's parents for the publication of this case report and for the use of images. A copy of the written consent is available for the Editor-in-Chief of this journal.

## Competing interests

The authors declare that they have no competing interests.

## Authors' contributions

SK and AO carried out the operation, and prepared the manuscript. AS participated in operation part and carried out the preparation of the figures. All authors read and approved the final manuscript.

## Footnotes

Migration of ventriculoperitoneal shunt. Karapolat et al.

## References

[B1] Taub E, Lavyne MH (1994). Thoracic complications of ventriculoperitoneal shunts: case report and review of the literature. Neurosurgery.

[B2] Akyuz M, Ucar T, Goksu E (2004). A thoracic complication of ventriculoperitoneal shunt: symptomatic hydrothorax from intrathoracic migration of a ventriculoperitoneal shunt catheter. Br J Neurosurg.

[B3] Savolaine ER, Khimji T (1991). Ventriculoperitoneal shunt failure resulting from complications of the thoracic segment of the shunt catheter. Case report. Clin Imaging.

[B4] Rahimi Rad MH, Mirzaagazadeh J, Ansarin K (2007). Supradiaphragmatic and transdiaphragmatic intrathoracic migration of a ventriculoperitoneal shunt catheter. Hong Kong Med J.

[B5] Doh JW, Bae HG, Lee KS, Yun IG, Byun BJ (1995). Hydrothorax from intrathoracic migration of a ventriculoperitoneal shunt catheter. Surg Neurol.

